# Systemic Effects of Vascular Photobiomodulation on Early Macrophage Recruitment During Skeletal Muscle Repair

**DOI:** 10.1002/jbio.202500368

**Published:** 2025-10-29

**Authors:** Raquel Lira Ortiz da Silva, Tainá Caroline dos Santos Malavazzi, Aline Souza Silva, Sandra Kalil Bussadori, Anna Carolina Ratto Tempestini Horliana, Fabio Daumas Nunes, Kristianne Porta Santos Fernandes, Maria Fernanda Setubal Destro Rodrigues, Raquel Agnelli Mesquita‐Ferrari

**Affiliations:** ^1^ Postgraduate Program in Biophotonics Medicine Universidade Nove de Julho (UNINOVE) Sao Paulo Sao Paulo Brazil; ^2^ Department of Oral Pathology, School of Dentistry University of São Paulo (FOUSP) São Paulo Sao Paulo Brazil; ^3^ Postgraduate Program in Rehabilitation Sciences Universidade Nove de Julho (UNINOVE) Sao Paulo Sao Paulo Brazil

**Keywords:** cryoinjury, low‐level laser therapy, macrophages, muscle repair, vascular photobiomodulation

## Abstract

Muscle injuries involve a complex inflammatory response in which macrophages are essential for proper tissue repair. Dysregulation of this process can impair regeneration. This study investigated the effects of vascular photobiomodulation (VPBM), a non‐invasive therapy applied via the caudal vein/artery, on macrophage dynamics in acute skeletal muscle injury. Wistar rats were divided into Control, Injury, Pre‐VPBM+Injury, and Injury+Post‐VPBM groups. VPBM (780 nm, 40 mW, 3.2 J) was applied preventively or therapeutically. Animals were euthanized on days 1, 2, 5, and 7 post‐injury for CD68^+^ and CD206^+^ immunostaining. CD68^+^ cells peaked on day 1 in the Pre‐VPBM+Injury group, while the Injury group maintained higher levels at day 7. CD206^+^ cells were elevated early in both VPBM groups, especially Pre‐VPBM. On day 7, CD206^+^ remained higher in the Injury group, indicating delayed resolution. In summary, the study demonstrates that preventive VPBM application accelerates macrophage recruitment and promotes a pro‐regenerative inflammatory response.

## Graphical abstract

Preventive vascular photobiomodulation accelerates macrophage recruitment and enhances pro‐regenerative inflammation after skeletal muscle injury, suggesting its potential to improve tissue repair through timely modulation of the immune response.

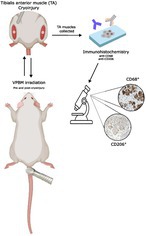

## 1 Introduction

Skeletal striated muscle is an abundant tissue in the human body, responsible for mobility and force generation. It is composed of muscle fibers, blood vessels, nerves, and extracellular matrix [1, 2, 3].

Although skeletal muscle has an inherent capacity for adaptation and regeneration, this process can be compromised when the injury becomes chronic due to the exacerbation or prolongation of the inflammatory and repair phases [1, 4]. Skeletal muscle injuries can occur through various mechanisms, regardless of an individual's physical condition. However, the risk increases when the muscle is pushed to its functional limits, as commonly observed in high‐performance athletes [1, 4, 5].

During the regeneration process, cytokines and chemotactic molecules are initially released, leading to the activation of satellite cells (SCs), which are key to the tissue's high regenerative capacity—while inflammatory cells are simultaneously recruited to the injury site [3, 6].

Neutrophils (elastase^+^) arrive within the first few hours and remain the predominant population for up to 24 h. After this period, the presence of cytokines such as IFN‐γ and TNF‐α triggers the recruitment of macrophages, primarily of the pro‐inflammatory M1 phenotype (CD68^+^) [1, 7]. These macrophages perform phagocytosis and release pro‐inflammatory mediators such as IL‐1β, IL‐6, TNF‐α, VEGF, and chemokines. Around the third day, macrophages polarize into the anti‐inflammatory M2 phenotype (CD206^+^), which releases cytokines such as VEGFA, TGF‐β, and IL‐10, leading to inflammation resolution, tissue repair, collagen synthesis, and remodeling [6, 8]. However, prolonged M1 macrophage presence at the injury site may cause tissue damage, while persistent M2 macrophages can contribute to tissue fibrosis [6, 8].

In this context, photobiomodulation (PBM) has emerged as a crucial tool for modulating these processes through the use of non‐ionizing light sources such as low‐level laser therapy (LLLT) or light‐emitting diodes (LEDs) [9, 10, 11, 12]. Studies demonstrate that, in addition to modulating the inflammatory response, PBM applied over the injured muscle tissue (LPBM) can enhance tissue repair, provide analgesia, regulate collagen synthesis and organization, and reduce edema [13, 14, 15]. By analyzing different aspects of muscle repair, including pre‐ and post‐injury PBM applications, Ribeiro et al. (2015) observed a reduction in myonecrosis and inflammatory infiltration, while Junior et al. (2020) and Souza et al. (2018) detailed the beneficial effects of LPBM in modulating M1 and M2 macrophage recruitment, which are crucial for inflammation resolution [16, 17, 18].

In this context, another form of PBM application, defined as vascular photobiomodulation (VPBM), enables non‐invasive blood irradiation by delivering light to the components of the circulating blood through the skin overlying veins or arteries [19, 20, 21]. The distal application with VPBM induces systemic immunomodulatory effects by irradiating blood cells that later migrate to and influence the injury site and muscle repair. Studies by Lopez et al. (2022) and Malavazzi et al. (2024) employing VPBM demonstrated significant modulation of inflammatory mediators, alongside reductions in muscle injury biomarkers, including creatine kinase (CK), aspartate aminotransferase (AST), and lactate [22, 23]. Importantly, prophylactic application resulted in improved histopathological outcomes, characterized by decreased myonecrosis and inflammatory cell infiltration, as well as increased neovascularization and regeneration of muscle fibers [22, 23].

The advantages of a systemic, single‐dose VPBM protocol include enhanced practicality and compliance, benefiting both patients and healthcare providers. This approach is particularly advantageous for high‐performance athletes, who frequently experience musculoskeletal injuries due to the high physical demands of training and competition, and for whom accelerated recovery is essential to minimize downtime.

Accordingly, the present study aimed to investigate the effects of VPBM on the recruitment of inflammatory cells to injured muscle tissue, with a specific focus on its temporal impact on distinct macrophage phenotypes—namely, pro‐inflammatory (CD68^+^) and anti‐inflammatory/repair‐associated (CD206^+^) subsets. This evaluation provides insight into the immunomodulatory dynamics elicited by systemic photobiomodulation and its potential to influence key cellular events involved in muscle regeneration.

## 2 Materials and Methods

The study was approved by the Animal Research Ethics Committee of the Universidade Nove de Julho (UNINOVE) under protocol number CEUA 1273010623. All procedures were conducted in accordance with the ethical standards and guidelines outlined by the National Council for the Control of Animal Experimentation (CONCEA).

A total of 65 adult male Wistar rats ( *Rattus norvegicus* , var. albinus, Rodentia, Mammalia), aged 12 weeks, were utilized in this study. Animals were housed in the UNINOVE animal facility under controlled environmental conditions: temperature maintained between 22°C and 25°C, relative humidity at 40%, and a 12:12‐h light/dark photoperiod. Rats were housed in standard polypropylene cages and received ad libitum access to commercial rodent chow (NUTRILAB CR‐1) and filtered water throughout the experimental period.

Animals were randomly allocated into four experimental groups:

Group 1—Control: No intervention (*n* = 5).

Group 2—Injury: Cryoinjury to the tibialis anterior (TA) muscle (*n* = 20).

Group 3—Pre‐VPBM + Injury: VPBM applied 24 h before cryoinjury (*n* = 20).

Group 4—Injury + Post‐VPBM: Cryoinjury followed by VPBM 2 h post‐injury (*n* = 20).

Animals in Groups 2, 3, and 4 were euthanized at 1‐, 2‐, 5‐, and 7‐days post‐injury (*n* = 5 per time point), while the control group was euthanized independently of the experimental timeline.

### 2.1 Muscle Injury Induction: Surgical Procedure

Cryoinjury, a widely utilized technique for the controlled induction of skeletal muscle damage, was performed using a metal rod cooled in liquid nitrogen, as per established protocols [24, 25, 26, 27]. The animals were weighed and anesthetized intraperitoneally with ketamine HCl (1 mL/kg) and xylazine (2%), after which the tibialis anterior (TA) muscle in both hind limbs was surgically exposed. The metal rod, pre‐cooled in liquid nitrogen for 30 s, was applied to the muscle for two cycles of 10 s each.

Postoperative pain management involved subcutaneous administration of dipyrone (50 mg/kg) and 2% tramadol hydrochloride (5 mg/kg) every 8 h for three consecutive days.

### 2.2 Photobiomodulation

The VPBM procedure was performed using a Gallium‐Aluminum‐Arsenide (AlGaAs) diode laser, model Twin‐Laser (MM Optics, São Carlos, SP, Brazil). Irradiation was directed at the caudal vein/artery, following the parameters outlined in Table 1. In groups subjected to VPBM prior to injury, treatment was administered 24 h before cryoinjury induction [22, 23]. For groups receiving VPBM after injury, irradiation began two hours after the surgical procedure and was repeated every 24 h [22, 23, 25, 28].

**TABLE 1:** VPBM dosimetric parameters.

Wavelength [nm]	780
Operating mode	Continuous
Power [mW]	40
Aperture diameter [cm]	0.23
Irradiance at aperture [W/cm^2^]	1
Beam area [cm^2^]	0.04
Exposure time [s]	80
Radiant exposure [J/cm^2^]	80
Number of points irradiated	1
Total irradiated energy [J]	3.2
Application technique	Direct contact

The experimental protocol included 1, 2, 5, and 7 treatment sessions, corresponding to evaluation time points at 1, 2, 5, and 7 days, respectively, until the animals were euthanized.

At the beginning and conclusion of the experimental procedure, the laser's light emission power was measured using a Laser Check power meter (MM Optics, São Carlos, SP, Brazil).

### 2.3 Euthanasia and Material Collection

**FIGURE 1:**
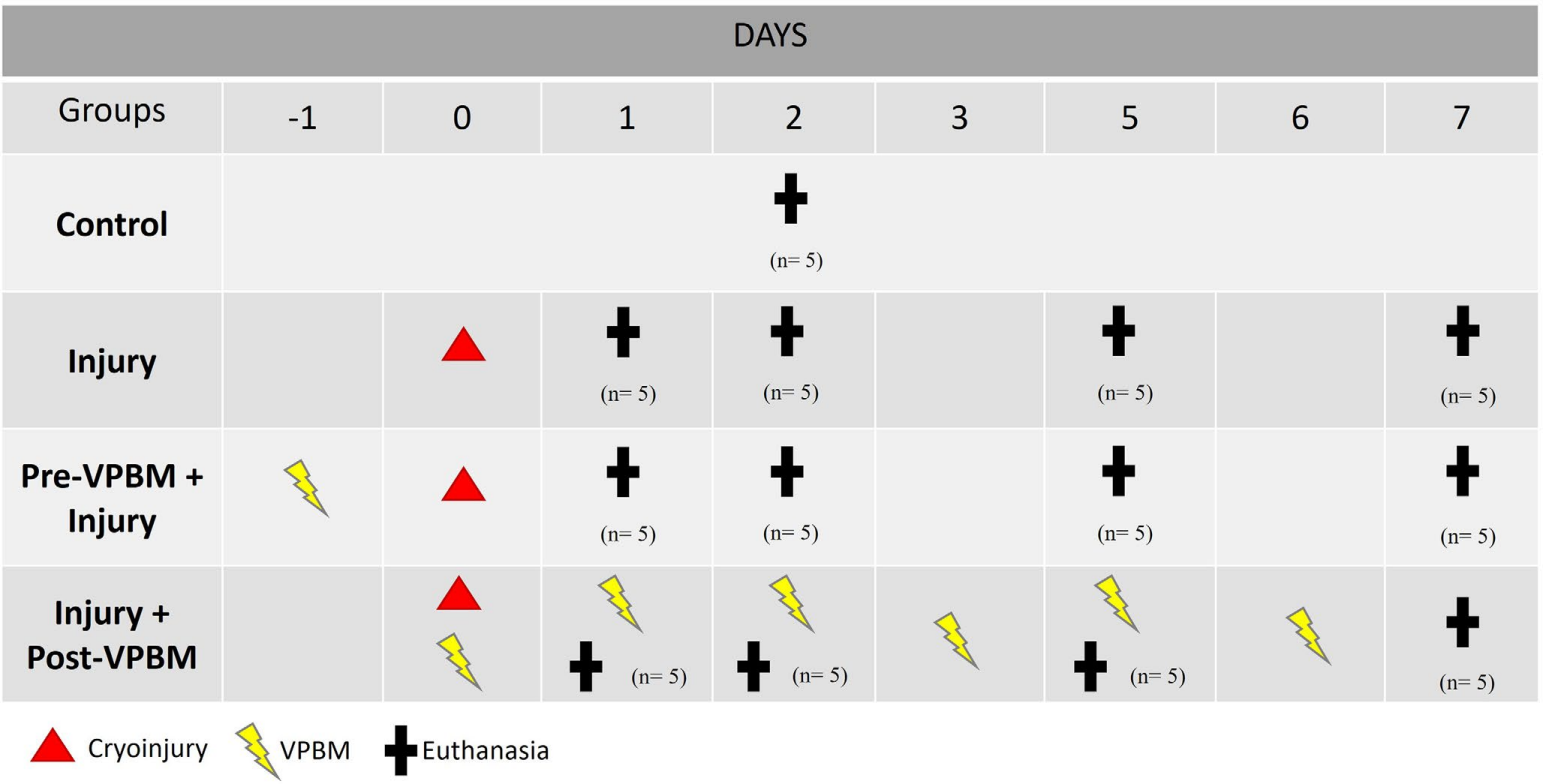
Timeline referring to the experimental periods and the processes of cryoinjury, VPBM, and euthanasia.

At the end of each experimental period (1, 2, 5, and 7 days), the animals were euthanized by an anesthetic overdose. Subsequently, the TA muscles from both hind limbs were excised and immediately fixed in 10% buffered formalin prepared in 1X PBS (pH 7.4) for 24 h. The tissues were then processed and embedded in paraffin according to standard histological preparation protocols (Figure 1).

### 2.4 Immunohistochemistry

Histological sections were deparaffinized, rehydrated, and subjected to antigen retrieval and endogenous peroxidase blocking using specific reagents from the Dako EnVision FLEX system (K8024). Sections were then incubated overnight (22 h) with primary anti‐CD68 (1:100, No. GTX41868, GenTex) and anti‐CD206 (1:150, CD206/MRC1 (E6T5J) XP Rabbit mAb #24595, Cell Signaling). The reaction was followed by incubation with the secondary antibody (EnVision FLEX/HRP) for 20 min and visualization using 3,3′‐diaminobenzidine (DAB) as the chromogen. Counterstaining was performed with Mayer's hematoxylin, and the slides were dehydrated and mounted for analysis.

The quantitative analysis was conducted using images acquired with a Zeiss Axioplan 2 microscope equipped with a 40× objective, yielding a total magnification of 400×. For each experimental group and time point, three samples were analyzed. From each slide, five randomly selected fields were imaged, and positively stained cells for CD68 and CD206 were manually counted using ImageJ software with a dedicated plug‐in. The analyses were performed in a randomized manner. Different evaluators were responsible for image acquisition and labeling, while a second evaluator performed the counting of positive cells for the different markers. Elsewhere, the animals were identified by random numbers, and group allocation analysis was performed in a blinded manner.

### 2.5 Statistical Analysis

Statistical analyses were performed using GraphPad Prism version 8.0.1 (San Diego, CA, USA). Data normality was assessed using the Kolmogorov–Smirnov and Shapiro–Wilk tests. Group comparisons were conducted using one‐way ANOVA followed by Tukey's post hoc test. A significant level of α = 5% was adopted.

## 3 Results

### 3.1 CD68 ^+^ Macrophage Dynamics Following VPBM in Injured Muscle

**FIGURE 2:**
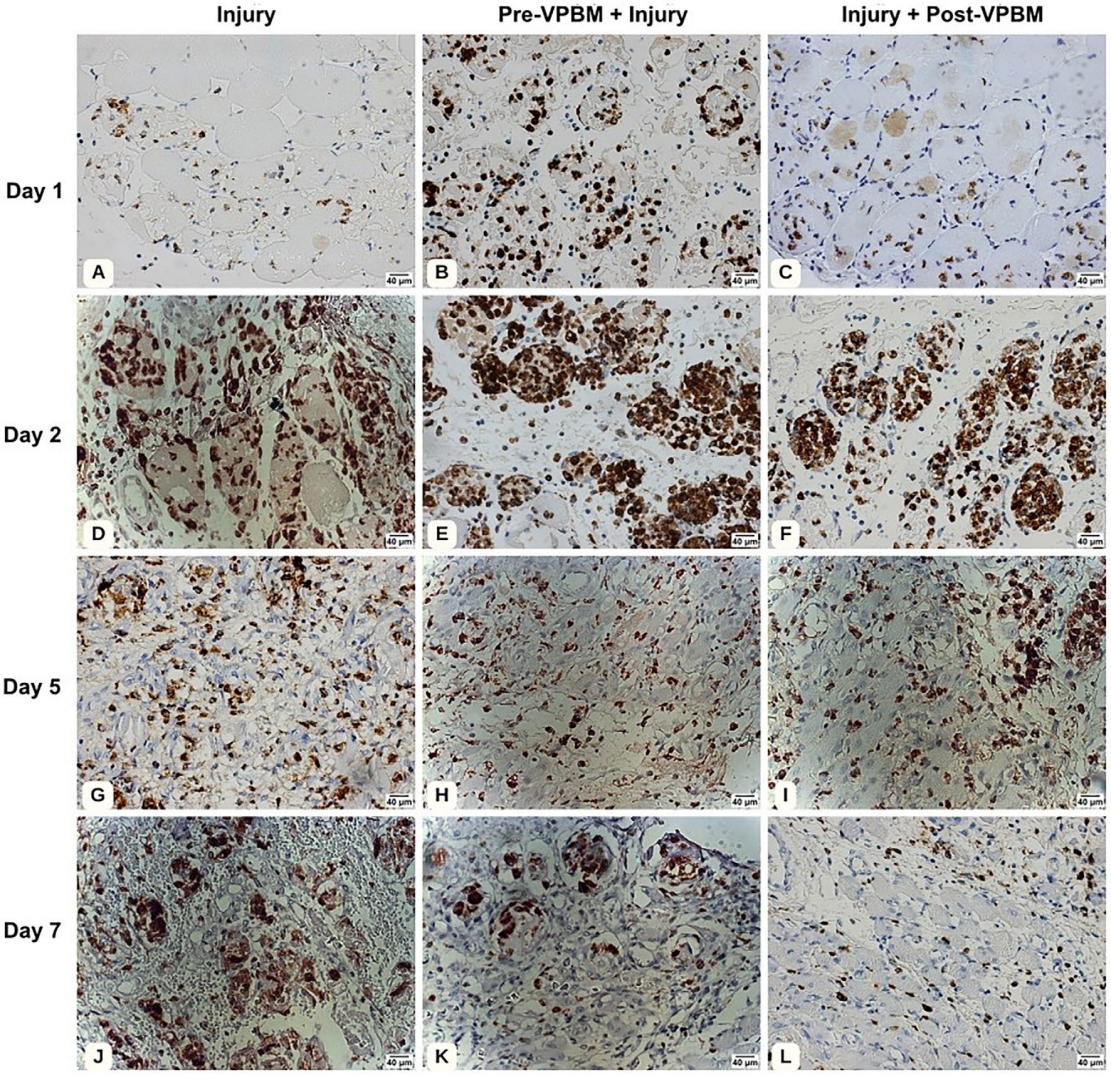
Representative photomicrographs of CD68^+^ immunostaining in tibialis anterior muscle sections at days 1, 2, 5, and 7. Injury (A, D, G, J), Pre‐VPBM + Injury (B, E, H, K), and Injury + Post‐VPBM (C, F, I, L).

The Injury group shows markedly increased CD68^+^ immunoreactivity at days 2 and 5, consistent with the peak and maintenance of the inflammatory response, as confirmed by quantitative analysis (Figure 1). The Pre‐VPBM + Injury group presents enhanced staining on day 1, with CD68^+^ levels exceeding those in the Injury group, suggesting a priming effect. However, by day 5, CD68^+^ cell numbers remain elevated and comparable to the Injury group. The Injury + Post‐VPBM group exhibits reduced CD68^+^ staining at days 5 and 7, in agreement with the quantitative data showing statistically significant decreases in CD68^+^ cell counts compared to the Injury group (Figure 2).

**FIGURE 3:**
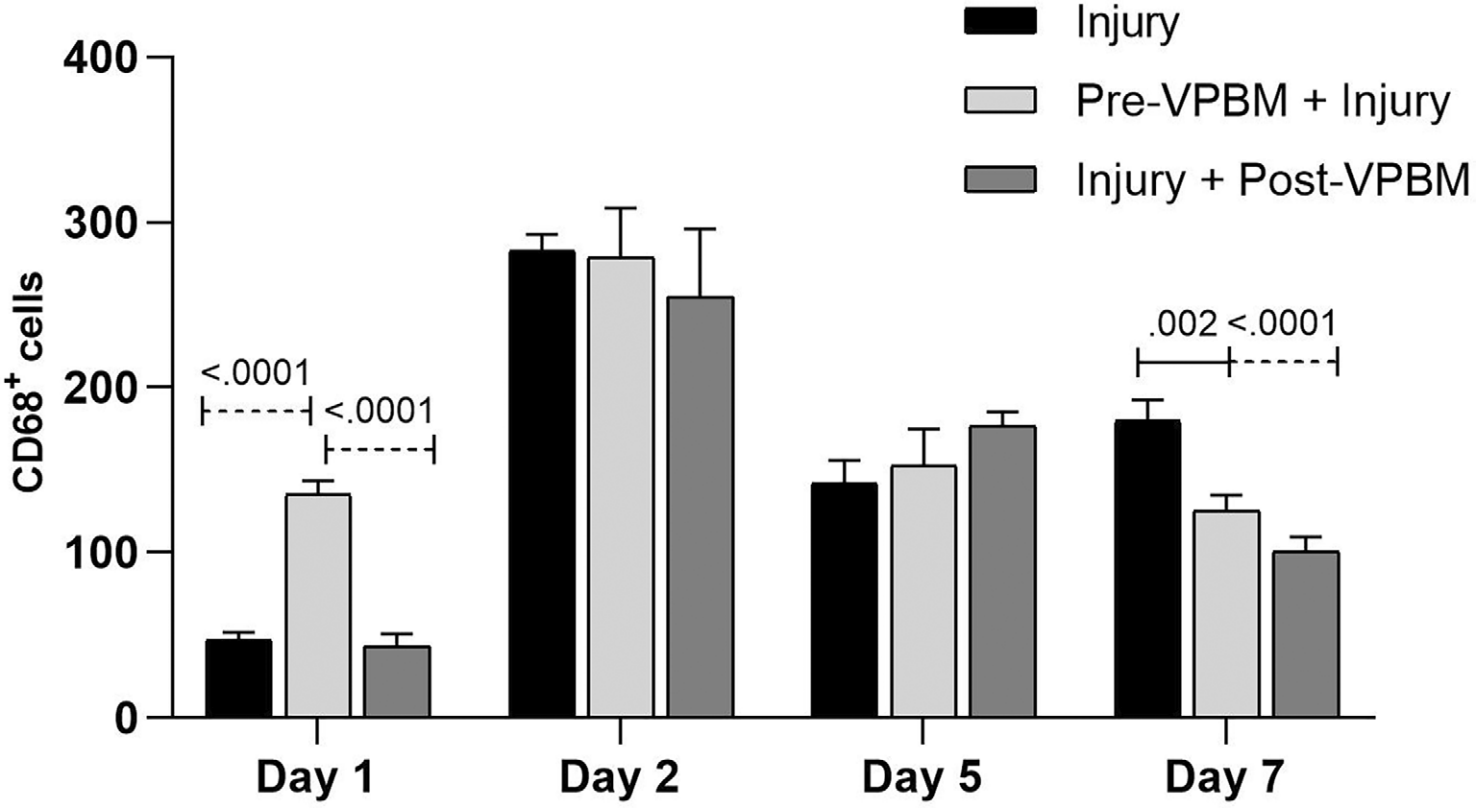
Quantification of CD68^+^ macrophages (pro‐inflammatory phenotype) in the tibialis anterior muscle at different time points following cryoinjury (days 1, 2, 5, and 7). Data are presented for the following groups: Injury, Pre‐VPBM+Injury, and Injury+Post‐VPBM. Results are expressed as mean ± SEM (ANOVA/Tukey).

Over the experimental timeline, the quantification of CD68^+^ cells showed distinct temporal patterns among the treatment groups (Figure 3). On day 1, the Pre‐VPBM + Injury group presented a significantly higher number of CD68^+^ cells compared to both the Injury (*p* < 0.05) and Injury + Post‐VPBM groups (*p* < 0.01), indicating an early increase in pro‐inflammatory macrophage infiltration. On day 2, CD68^+^ cell counts were elevated in all groups, with no statistically significant differences observed among them. On day 5, CD68^+^ cell numbers declined across all conditions, but without significant intergroup differences. By day 7, the Injury group exhibited significantly higher CD68^+^ cell counts compared to the Pre‐VPBM + Injury group (*p* < 0.05) and the Injury + Post‐VPBM group (*p* < 0.001), with no statistical difference between the two VPBM‐treated groups (Figure 3).

### 3.2 CD206 ^+^ Macrophage Response Over Time in VPBM‐Treated Muscle Injury

**FIGURE 4:**
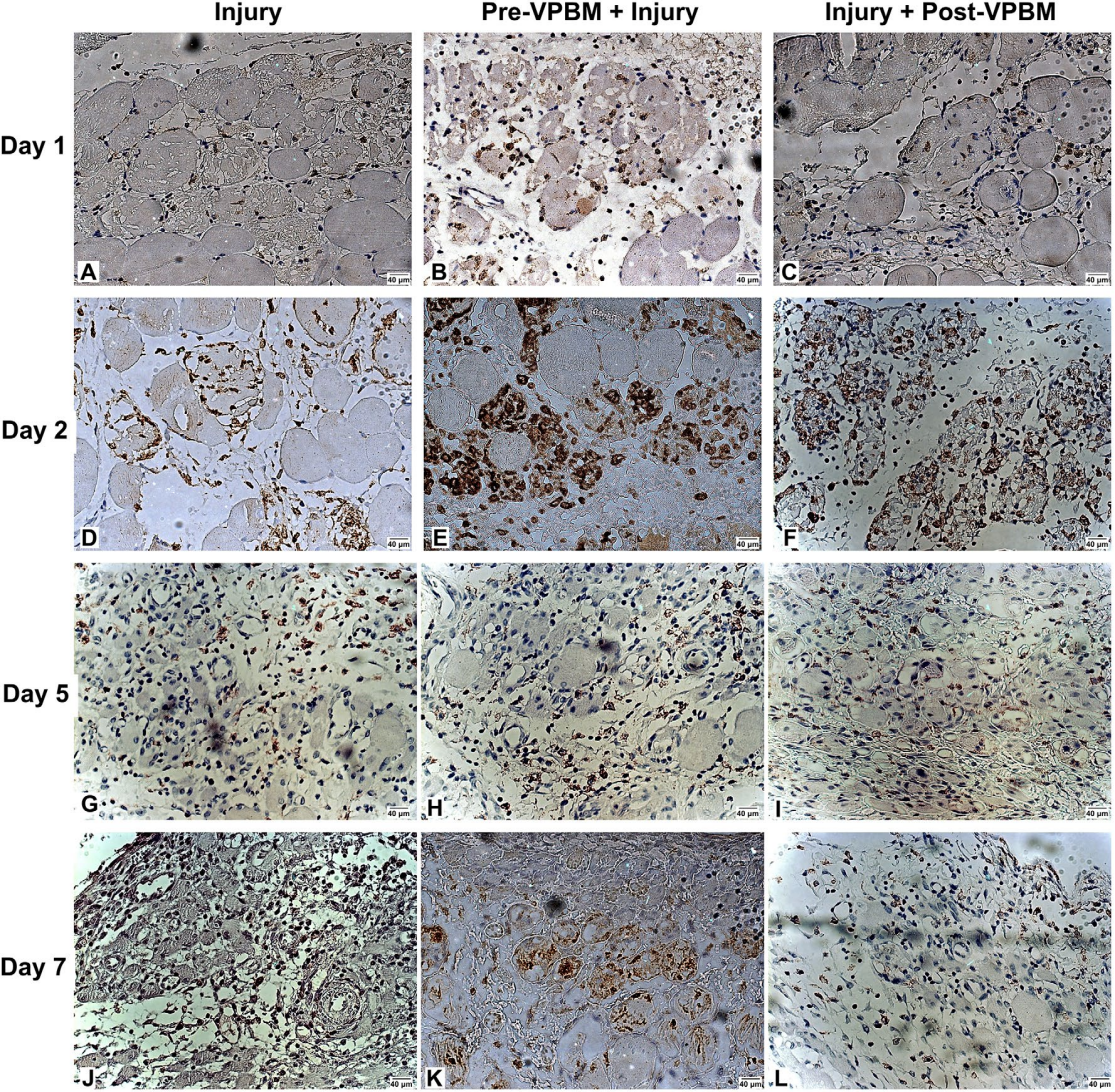
Representative photomicrographs of CD206^+^ immunostaining in tibialis anterior muscle sections at days 1, 2, 5, and 7. Experimental groups: Injury (A, D, G, J), pre‐VPBM + Injury (B, E, H, K), and Injury + post‐VPBM (C, F, I, L).

Considering the CD206 immunostaining, the injury group shows minimal staining at days 1 and 2, with a slight increase observed at days 5 and 7, suggesting a delayed transition toward a reparative macrophage phenotype during muscle regeneration (Figure 4). In contrast, the pre‐VPBM + Injury group demonstrates more prominent CD206^+^ staining starting from day 2, with sustained expression at days 5 and 7, indicating an earlier onset of anti‐inflammatory response, potentially due to a priming effect induced by PBM. The Injury + post‐VPBM group exhibits increased CD206^+^ staining primarily at days 5 and 7, with stronger intensity than the injury group (Figure 4).

**FIGURE 5:**
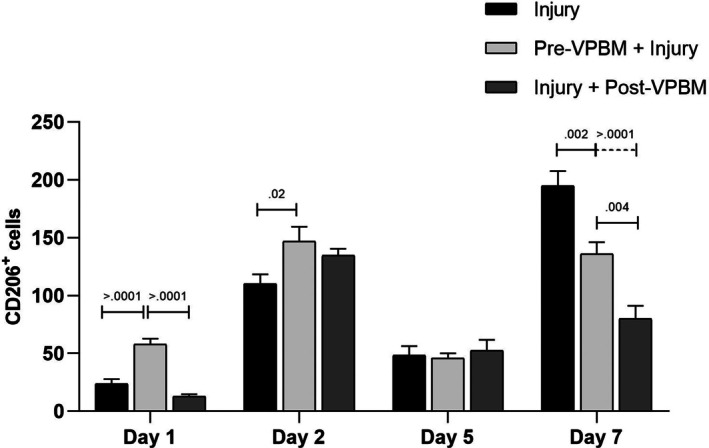
Quantification of CD206^+^ macrophages (anti‐inflammatory/repair phenotype) in the tibialis anterior muscle at different time points following cryoinjury (days 1, 2, 5, and 7). Data are presented for the following groups: Injury, Pre‐VPBM+Injury, and Injury+Post‐VPBM. Results are expressed as mean ± SEM (ANOVA/Tukey).

Over the experimental timeline, the quantification of CD206^+^ cells revealed distinct temporal patterns among the treatment groups (Figure 5). On day 1, both Pre‐VPBM + Injury and Injury + Post VPBM groups exhibited a significant increase in CD206^+^ cells compared to the Injury group (*p* < 0.0001), with the highest values observed in the Pre‐VPBM + Injury group. On day 2, CD206^+^ cell counts remained elevated in the Pre‐VPBM + Injury group, which was significantly different from the Injury group (*p* = 0.0225), while no significant difference was observed between the Pre‐VPBM + Injury and Injury + Post‐VPBM groups (*p* = 0.6322). On day 5, CD206^+^ cell numbers declined in all groups, with no statistically significant differences among them. By day 7, the Injury group showed a significantly higher number of CD206^+^ cells compared to both Pre‐VPBM + Injury (*p* = 0.0053) and Injury + Post VPBM (*p* < 0.0001), with a significant difference also observed between the VPBM‐treated groups (*p* = 0.0482) (Figure 5).

## 4 Discussion

Although the mechanism of action of VPBM is closely linked to systemic inflammatory mediators present in the bloodstream, the literature still lacks studies assessing the effects of vascular, non‐invasive, and preventive PBM on the recruitment of inflammatory cells, particularly in skeletal muscle injury models. This study addresses that gap by evaluating how VPBM modulates the temporal dynamics of macrophage subtypes—CD68^+^ (M1/pro‐inflammatory) and CD206^+^ (M2/anti‐inflammatory)—in the injured muscle microenvironment.

Circulating monocytes typically differentiate into macrophages and migrate to injury sites within 48 h [1, 8, 29], a timeline consistent with the observed CD68^+^ macrophage peak on day 2 in the Injury and Injury+Post‐VPBM groups. However, in the Pre‐VPBM+Injury group, this response was significantly anticipated, with increased CD68^+^ cell counts already present on day 1, suggesting that preventive VPBM promotes earlier monocyte recruitment and differentiation at the injury site.

Junior et al. (2019) investigated local PBM (780 nm, 40 mW, 3.2 J) in elderly rats and reported CD68^+^ macrophage recruitment peaking on day 3 post‐injury [17]. Although the time of evaluations differs, the current findings align in demonstrating the capacity of PBM to modulate macrophage dynamics. Notably, VPBM in this study induced a CD68^+^ increase as early as day 1 in the Pre‐VPBM + Injury group and on day 2 in the Injury + Post‐VPBM group, reinforcing that both timing and mode of PBM application (vascular versus local) are critical modulators of the inflammatory response.

Souza et al. (2018), using local PBM (LPBM) with red (660 nm, 70 mW, 8 J) and infrared (780 nm, 70 mW, 8 J) wavelengths, found a significant reduction in CD68^+^ macrophage counts two days post‐injury in a model of acute muscle damage [18]. These findings diverge from the current results, where a peak in CD68^+^ cells was observed at the same time point in the Injury + Post‐VPBM group. This discrepancy may be attributed to differences in PBM delivery methods. LPBM acts directly on the injured tissue, modulating the local microenvironment, whereas VPBM targets circulating blood components, potentially priming immune cells before they migrate to the injury site [20, 21]. Furthermore, the energy dose and irradiated area differ substantially, which could influence cellular responses. Notably, despite these differences, both studies converge by day 7, showing reduced CD68^+^ levels, consistent with the resolution of the inflammatory phase. Regarding M2 macrophages (CD206^+^), the present study demonstrated earlier and more robust recruitment in both VPBM‐treated groups. On day 1, CD206^+^ counts were significantly elevated compared to the Injury group, particularly in the Pre‐VPBM + Injury group. Peak expression occurred on day 2 in both Pre‐VPBM + Injury and Injury + Post‐VPBM groups, followed by a marked decline by day 7. This accelerated shift toward an anti‐inflammatory profile suggests that VPBM—especially when applied preventively—may shorten the duration of the pro‐inflammatory phase, facilitating a more regulated and efficient tissue repair process.

These findings are supported by Jahani‐Sherafat et al. (2024) [30], who reported increased CD206 expression following PBM in an in vitro inflammatory model, and by Zhang et al. (2019) [31], who observed similar effects in spinal cord injury models. Likewise, Junior et al. (2019) [17] found increased M2 macrophages after day 3, highlighting VPBM's unique capacity to induce this phenotype earlier—day 1 in this study—especially with preventive application.

Zagatto et al. (2016) demonstrated that local PBM modulates muscle damage markers (CK, LDH) in athletes, although without significant cytokine changes, possibly due to small irradiated volumes [32]. In contrast, Malavazzi et al. (2024) reported increased TNF‐α and IL‐1β levels after VPBM, consistent with the early pro‐inflammatory response seen in the Pre‐VPBM+Injury group [22]. This suggests that VPBM enhances systemic monocyte mobilization and extravascular recruitment, impacting macrophage polarization.

Collectively, the photobiomodulation (PBM) delivery route—local (LPBM) versus vascular (VPBM)—exerts distinct modulatory effects on the inflammatory cascade, primarily due to differences in the primary photonic interaction targets [20]. LPBM directly irradiates the injured tissue, acting on resident cells, infiltrating leukocytes, and the local microenvironment, thereby influencing cellular metabolism, reactive oxygen species (ROS) levels, and local cytokine expression [18, 28, 33]. Conversely, VPBM targets circulating blood components—such as monocytes, lymphocytes, and vascular endothelial cells—potentially priming these cells before their homing to sites of tissue injury. This systemic photobiological interaction modulates leukocyte‐endothelial interactions, inflammatory mediator release, and macrophage differentiation kinetics [21, 22, 23, 34, 35, 36].

The main targets of VPBM are circulating cells within the intravascular environment, including red blood cells, leukocytes, lymphocytes, and platelets. Among these, the porphyrins in red blood cells play a central role by absorbing light energy, which increases membrane permeability and enhances cytoplasmic dynamics [22]. This process contributes to heme group formation, essential for oxygen transport. Such mechanisms suggest that VPBM can provide bioenergetic support to circulating cells and strengthen their defense capacity [20].

These mechanistic distinctions are reflected in the divergent temporal patterns of macrophage subtype recruitment observed in the present study. VPBM, especially when administered preventively, accelerated the infiltration of both CD68^+^ (classically activated, pro‐inflammatory) and CD206^+^ (alternatively activated, anti‐inflammatory/pro‐regenerative) macrophages. This anticipatory recruitment appears to shorten the pro‐inflammatory phase and promote a more rapid transition toward tissue remodeling. Such temporal reprogramming may prevent prolonged inflammation, mitigate secondary damage to muscle fibers, and enhance the resolution of inflammation, ultimately contributing to more favorable regenerative outcomes [22].

From a translational standpoint, the present findings highlight the therapeutic potential of VPBM as a systemic, non‐invasive modality to modulate immune cell dynamics following skeletal muscle injury. Particularly in contexts demanding expedited recovery—such as in elite athletes—VPBM may serve as an adjunct strategy to attenuate edema, nociceptive signaling, and tissue degradation. By promoting early polarization toward the M2 macrophage phenotype and fostering a pro‐regenerative microenvironment, VPBM could optimize skeletal muscle repair and reduce the time required to reintegrate into physical activity or training regimens.

## 5 Conclusion

In conclusion, VPBM was able to modulate the temporal recruitment of M1 and M2 macrophages, with more pronounced effects in the preventive protocol, promoting an accelerated transition toward a pro‐regenerative profile. These findings highlight its potential in enhancing muscle repair and support further research in populations with impaired healing, such as those with obesity or diabetes. Standardization of dosimetric parameters remains essential to ensure clinical applicability and therapeutic consistency.

## Author Contributions

**Raquel Lira Ortiz da Silva:** conceptualization, methodology, investigation, writing – original draft preparation, visualization, data curation, formal analysis, analysis of results. **Tainá Caroline dos Santos Malavazzi:** methodology, investigation, visualization, data curation, writing – original draft preparation and visualization, analysis of results. **Aline Souza Silva:** investigation, writing – original draft preparation, visualization, data curation, formal analysis. **Sandra Kalil Bussadori:** project administration, validation and writing – reviewing and editing. **Anna Carolina Ratto Tempestini Horliana:** project administration, validation and writing – reviewing and editing. **Fabio Daumas Nunes:** project administration, data curation, validation, writing – reviewing and editing. **Kristianne Porta Santos Fernandes:** project administration, data curation, validation, writing – reviewing and editing. **Maria Fernanda Setubal Destro Rodrigues:** project administration, methodology, validation, writing – reviewing and editing. **Raquel Agnelli Mesquita‐Ferrari:** resources, visualization, data curation, validation, writing – reviewing and editing and supervision.

## Ethics Statement

This study received approval from the local animal research ethics committee—Nove de Julho University (certificate number: 1273010623).

## Conflicts of Interest

The authors declare no conflicts of interest.

## Data Availability

The data that support the findings of this study are available from the corresponding author upon reasonable request.
